# Pulsed Electric Fields-Assisted Extraction of Valuable Compounds From Arthrospira Platensis: Effect of Pulse Polarity and Mild Heating

**DOI:** 10.3389/fbioe.2020.551272

**Published:** 2020-09-04

**Authors:** Daniele Carullo, Gianpiero Pataro, Francesco Donsì, Giovanna Ferrari

**Affiliations:** ^1^Department of Industrial Engineering, University of Salerno, Fisciano, Italy; ^2^ProdAl Scarl – University of Salerno, Fisciano, Italy

**Keywords:** *Arhtrospira platensis*, pulsed electric fields, pulse polarity, temperature, extraction, water-soluble compounds, energy efficiency

## Abstract

The present study aimed to investigate the effect of the main pulsed electric field (PEF) process parameters on the cell damages of *A. platensis* microalgae and the extractability of valuable compounds [water-soluble proteins (WSP), C-phycocyanin (C-PC), and carbohydrates (CH)]. Aqueous microalgae suspensions (2%, w/w) were PEF-treated at variable field strength (*E* = 10, 20, 30 kV/cm), total specific energy (*W*_T_ = 20, 60, 100 kJ/kg_susp_), and inlet temperature (25, 35, 45°C), with either monopolar or bipolar square wave pulses (5 μs of width, delay time between pulses of opposite polarities = 1, 5, 10, 20 μs), prior to extraction with water at room temperature (25°C) for up to 3 h. High-pressure homogenization (HPH) treatment (*P* = 150 MPa, 3 passes) was used to achieve complete cell disruption to quantify the total extractable content of target intracellular compounds. Scanning electron microscopy (SEM) and optical microscopy analyses clearly showed that PEF merely electroporated the membranes of algae cell, without damaging the cell structure and forming cell debris. The application of PEF treatment (monopolar pulses, 20 kV/cm and 100 kJ/kg_susp_) at room temperature significantly enhanced the extraction yield of WSP [17.4% dry weight (DW)], CH (10.1% DW), and C-PC (2.1% DW), in comparison with the untreated samples. Bipolar pulses appeared less effective than monopolar pulses and led to extraction yields dependent on the delay time. Additionally, regardless of pulse polarity, a clear synergistic effect of the combined PEF (20 kV/cm and 100 kJ/kg_susp_)-temperature (35°C) treatment was detected, which enabled the extraction of up to 37.4% (w/w) of total WSP, 73.8% of total CH, and 73.7% of total C-PC. Remarkably, the PEF treatment enabled to obtain C-phycocyanin extract with higher purity than that obtained using HPH treatment. The results obtained in this work suggest that the application of PEF combined with mild heating could represent a suitable approach for the efficient recovery of water-soluble compounds microalgal biomass.

## Introduction

*Arthrospira platensis*, commonly known as spirulina, is a cyanobacterium widely used for biotechnological applications. It is a multicellular and filamentous blue-green alga with helical shape (trichomes of 50–500 μm in length, and 3–4 μm in width) and represents one of the richest sources of proteins of microbial origin [55–70% dry weight (DW)]; besides, it also contains significant amounts of carbohydrates (13–16% DW), lipids (6–10% DW), vitamins, and minerals (Lupatini et al., 2016). In particular, *A. platensis* is an excellent source of C-phycocyanin (C-PC), a water-soluble pigmented protein, which has gained importance in many applications in the food, cosmetic and pharmaceutical sectors, thanks to its blue color and its therapeutic properties, as well as for its potential use as a fluorescent marker in biomedical research (Fernández-Rojas et al., 2014). In *A. platensis*, C-PC serves as a light-harvesting pigment for the photosynthetic activity of this cyanobacteria, in which it is assembled, along with other phycobiliproteins, in the thylakoid membranes of chloroplast (Martinez et al., 2020), and may represent more than 20% of its dry weight (Martinez et al., 2017).

In general, the recovery of intracellular compounds of interest from algal biomass via conventional solvent extraction techniques is hampered by the presence of the rigid cell wall and membranes, which act as a barrier that greatly limits the penetration of the solvent into the cytoplasm and the diffusion of the solubilized intracellular compounds during the extraction process (Martinez et al., 2020). For these reasons, to recover a substantial amount of valuable compounds, the conventional extraction techniques may require the use of a large amount of solvent, long extraction time and relatively high temperature that may cause losses of labile compounds, as well as lead to the co-extraction of undesirable components (Günerken et al., 2015; Poojary et al., 2016). Moreover, the extraction process is often conducted upon drying of algae biomass, which requires a significant amount of energy and may cause thermal degradation of valuable compounds (Günerken et al., 2015; Golberg et al., 2016). In this regard, it has been reported that the initial amount of C-PC in *A. platensis* decreased by approximately 50% after the drying of the algae biomass (Martinez et al., 2017).

In light of these drawbacks of conventional solvent extraction methods, cell disruption pre-treatment of wet biomass that causes weakening or breakage of cell envelops is required to intensify the extractability of specific intracellular compounds with reduced energy consumption, while preserving or improving the quality (purity) of the extracts (Poojary et al., 2016). Among the cell disruption methods, freeze/thawing cycles, sonication, bead milling, and high-pressure homogenization (HPH) treatments have been widely studied as pre-treatment of either microalgae and cyanobacteria biomasses, due to their ability to induce complete cell disruption, which markedly increases the extraction yield of the components located within the algae cells. However, all these methods cause the non-selective release of intracellular compounds, with the concurrent dispersion of cell debris or other impurities into the extraction medium, hence decreasing the quality of the extracts and complicating the subsequent downstream purification operations (Aouir et al., 2015; Poojary et al., 2016; Martinez et al., 2017; Carullo et al., 2018; Jaeschke et al., 2019).

In this frame, pulsed electric fields (PEF) is considered to be a very promising technology for mild and scalable cell disruption of wet biomass, thus avoiding the need for energy-intensive drying and the consequent losses of labile compounds (Carullo et al., 2018). The technique consists of exposing fresh algae suspensions to repetitive high-intensity electric field pulses of short (of the order of μs) duration that cause the permeabilization of cell membranes by electroporation. This improves the efficiency of the conventional extraction process of valuable compounds from algae biomass, facilitating the penetration of the solvent into the cells and the selective release of intracellular matter (Poojary et al., 2016) without the formation of cell debris (Martinez et al., 2017; Phong et al., 2017; Carullo et al., 2018; Pataro et al., 2019). Recently, several studies have demonstrated the potential of PEF to intensify the extraction yield of target intracellular compounds, such as lipids, pigments, carbohydrates, and proteins, from different microalgae and cyanobacteria (Goettel et al., 2013; Zbinden et al., 2013; Grimi et al., 2014; Luengo et al., 2015; Parniakov et al., 2015a,b; Carullo et al., 2018; Geada et al., 2018; Silve et al., 2018), even though, to date, only a few works focused on *A. platensis* (Aouir et al., 2015; Martinez et al., 2017; Jaeschke et al., 2019).

However, it has been shown that the recovery of substantial amounts of compounds of relatively high molecular weight (e.g., protein) could require the application of intense PEF processing conditions (high field strengths and energy input), especially in the case of “hard-structured” microalgal cells (Postma et al., 2016; Pataro et al., 2019). Therefore, to reduce the operative costs and to maximize the extraction efficiency of high-added-value components, the use of PEF in a hurdle approach has been suggested. For example, several studies have demonstrated that the combination of PEF with moderate heating decreases the critical electric field required to cause electroporation in both microbial and algae cells, thus resulting in additive or synergistic effects in microbial inactivation or extraction of intracellular compounds (Saldaña et al., 2014; Timmermans et al., 2014; Luengo et al., 2015; Postma et al., 2016; Martinez et al., 2017). However, as per the literature survey, only Martinez et al. (2017) described the effect of temperature (10–40°C) on the release of C-PC during PEF treatment of *A. platensis*, using a batch chamber equipped with a temperature control system.

Additionally, the use of bipolar pulses, which appear to be more efficient than monopolar ones, could be also suggested to obtain the required permeabilization effect with less severe processing conditions or to achieve higher efficacy at the same treatment intensity. Nevertheless, very few works have dealt so far with the influence of pulse polarity on the extent of electroporation of biological cell, and only for microbial inactivation purposes (Chang, 1989; Qin et al., 1994; Beveridge et al., 2002; Evrendilek and Zhang, 2005).

This research aimed to assess the potential of the application of PEF, either as a stand-alone treatment or in a hurdle approach with moderate heating, for the intensification of the extraction of valuable compounds from wet *A. platensis* biomass using a continuous flow system. Specifically, the effect of field strength, energy input, pulse polarity, and inlet temperature of the algae biomass to the PEF chamber on the morphology of algae cells, as well as on the extractability of target intracellular compounds (e.g., water-soluble proteins, C-phycocyanin, and carbohydrates), was assessed.

## Materials and Methods

### Cultivation of Microalgae

Biomass of *A. platensis* (PCC 8005) was kindly supplied by ATI Biotech Srl, an algae producer located in Castel Baronia (Avellino, Italy). *A. platensis* was cultivated in open pond systems, in which a maximum biomass concentration of about 0.4% DW was achieved at the end of the exponential growth phase. After harvesting, the biomass was concentrated through a dewatering system consisting of vibrating screen filters, which allowed to increase the biomass concentration up to 12% DW. The microalgae paste was subsequently packed in polyethylene bags and immediately transported in an expanded polystyrene (EPS) box under refrigerated conditions to the laboratory of ProdAl S.c.a.r.l. (University of Salerno, Fisciano, Italy), where it was stored at 4°C until use, within 2 days from the delivery date.

Before processing, the algae paste was diluted with distilled water up to a final concentration (C_x_) of 2% DW with an initial conductivity of about 2.7 mS/cm at 25°C (Conductivity-meter HI 9033, Hanna Instrument, Milan, Italy). The biomass concentration was assessed using the method described by Goettel et al. (2013), with the pellet being dried in a circulating air-drying oven for 24 h at 80°C.

### Pulsed Electric Fields System

PEF experiments were conducted in a bench-scale continuous flow PEF system previously described in detail by Postma et al. (2016) and Carullo et al. (2018). Briefly, it consisted of a peristaltic pump used to transfer the microalgal suspension through a stainless steel coiled tube submerged into a water heating bath used to control the inlet temperature to the treatment chamber. The latter consisted of two modules, each made of two co-linear treatment chambers, hydraulically connected in series, with an inner radius of 1.5 mm and a gap distance of 4 mm. The treatment chambers were connected to a high voltage pulsed power (20 kV–100 A) generator (Diversified Technology Inc., Bedford, WA, United States) able to deliver either monopolar or bipolar square wave pulses at different pulse width (1–10 μs), delay time between two consecutive pulses of opposite polarities (1–20 μs) and pulse repetition rate (1–1000 Hz). The maximum electric field intensity (E, in kV/cm) and total specific energy input (W_T_, in kJ/kg_susp_) were measured and calculated as reported in Postma et al. (2016). T-thermocouples (Tersid S.r.L, Milan, Italy) were used to measure the product temperature at the inlet and outlet of each module of the PEF chamber. The total residence time of the algae suspension in the PEF plant at each processing temperature was about 25 s.

### PEF Treatments

PEF treatments were carried out by pumping algae suspension (2% DW) from a feeding tank under stirring through the treatment chamber at a constant flow rate of 2 L/h. The operative pressure was about 1 bar. In all the experiments, the pulse width was fixed at 5 μs, while the electric field strength (E) and total specific energy input (W_T_) were set by varying the applied voltage and the pulse repetition frequency, respectively. The inlet biomass temperature (T_IN_) to the PEF treatment chamber was set at 25°C unless otherwise specified.

Three different experiments were carried out to investigate the effect of treatment intensity, pulse polarity, and inlet temperature on the cell damage and extraction efficiency of water-soluble compounds such as proteins, carbohydrates, and C-phycocyanins from *A. platensis* cells.

In the first set of experiments, a screening of the main electric parameters, namely field strength (10, 20, and 30 kV/cm) and energy input (20, 60, and 100 kJ/kg_susp_), on the release of water-soluble compounds was investigated. The specific energy input per pulse (W_p_) was equal to 0.95, 3.65, and 8.36 kJ/kg, when the field strength was set at 10, 20, and 30 kV/cm, respectively, and the number of pulses applied ranged between 2 and 105. The results of this first set of experiments were used to define the optimal PEF conditions (E_OPT_, W_T_,_OPT_), which enabled the achievement of the highest recovery yields with the minimum treatment severity.

**FIGURE 1:**
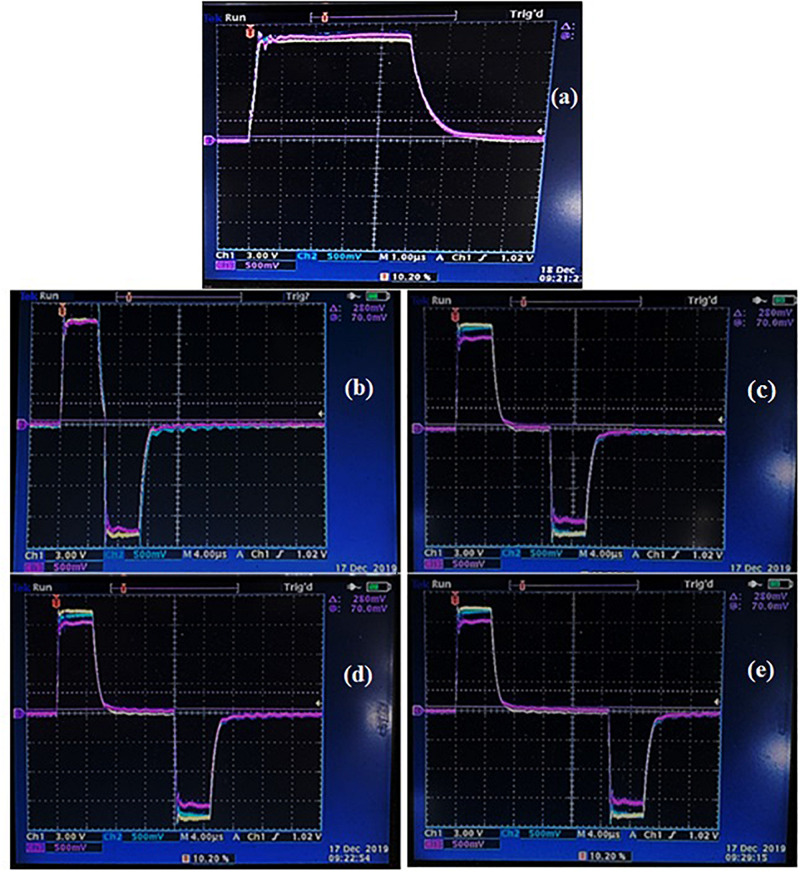
Typical voltage and current waveforms captured at the treatment chamber for either monopolar **(a)** or bipolar pulses at different delay time of 1 μs **(b)**, 5 μs **(c)**, 10 μs **(d)**, and 20 μs **(e)**. Reference color: yellow: voltage waveform; light blue: current waveform at the first module of the treatment chamber; purple: current waveform at the second module of the treatment chamber.

In the second set of experiments, a comparative study was carried out to investigate the effect of pulse polarity on the cell membrane permeabilization and extraction efficiency. Monopolar and bipolar pulses at different delay times (1, 5, 10, and 20 μs) were applied at the optimal field strength (E_OPT_) and energy input (W_T,__OPT_) defined during the first set of experiments. The typical voltage and current waveforms at the treatment chamber, for either monopolar or bipolar pulses at different delay times, are depicted in Figure 1.

Finally, the use of PEF treatments (E_OPT_, W_T,OPT_) applied using either monopolar or bipolar pulses in combination with mild heating treatment, achieved by raising the inlet biomass temperature (T_IN_) to the treatment chamber up to 25, 35, and 45°C, was investigated to highlight the existence of possible synergistic effects of the combined treatment in the recovery process of intracellular compounds. Temperatures higher than 45°C were not tested in this work to prevent any damage to proteins as well as degradation of C-PC (Chaiklahan et al., 2012).

Under the selected operating conditions, the maximum temperature increase of the samples, detected at the exit of the treatment chamber, never exceeded 10°C.

For the sake of comparison, untreated (control) samples of the same *A. platensis* suspension were pumped through the PEF plant with the heating bath set at 25, 35, or 45°C, but with the PEF generator switched off.

At the exit of the treatment chamber, untreated (control) and PEF treated algae suspensions were immediately collected in plastic tubes and placed in an ice-water bath to be rapidly cooled up to a final temperature of 25°C before undergoing to the aqueous extraction process.

### Water Extraction

Untreated and PEF treated samples of *A. platensis* suspensions were allowed to stand for 3 h at 25°C under gentle agitation (160 rpm) in an orbital incubator (Model S150, PBI International, Milan, Italy) to allow intracellular components to diffuse out of the cells. Preliminary tests revealed that these conditions were sufficient to achieve significant extraction yields of the target intracellular compounds (data not shown). After this resting time, the cell suspensions were centrifuged for 10 min at 5700 × g (PK121R model, ALC International, Cologno Monzese, IT) in order to separate the spent pellets from the supernatants. The latter were then transferred to fresh tubes and stored at −20°C until further analysis.

### Complete Cell Disruption by HPH Treatment

HPH treatment was used to induce full disruption of *A. platensis* cells to enable the quantification of the total content of target intracellular compounds. HPH treatments were carried out by using an in-house developed laboratory scale high-pressure homogenizer (Carullo et al., 2018). The *A. platensis* suspensions, at the same concentration as for PEF treatment tests (2% DW), were forced to pass through a 100 μm diameter orifice valve (model WS1973, Maximator JET GmbH, Schweinfurt, Germany) upon pressurization through an air-driven Haskel pump (model DXHF-683, EGAR S.r.l., Milan, Italy). According to preliminary tests (data not shown), the full disruption of *A. platensis* cells and, hence, the complete release of intracellular compounds, was achieved at a pressure drop (P) across the orifice of 150 MPa and after three homogenization passes (n_p_).

### Energy Analysis

To enable the comparison in terms of energy efficiency among the different investigated extraction processes (i.e., PEF, mild heating, combination of PEF and mild heating, and HPH), the energy consumed (EC) to extract 1 kg DW of target intracellular compounds, namely water-soluble proteins (WSP), C-phycocyanin (C-PC) and total carbohydrates (CH), from *A. platensis* cell suspension, was calculated according to Eqs. (1–3).

(1) $${\mathrm{EC}}_{\mathrm{PEF}}=\frac{W_{T,O\,P\,T}}{C_{x}\cdot 3600\cdot Y_{i}}$$

(2) $${\mathrm{EC}}_{\mathrm{HEATING}}=\frac{c_{P}\cdot \left(T_{I\,N}-T_{0}\right)}{C_{x}\cdot 3600\cdot Y_{i}}$$

(3) $${\mathrm{EC}}_{\mathrm{HPH}}=\frac{P\cdot n_{P}}{C_{x}\cdot \eta_{P\,U\,M\,P}\cdot 3600\cdot \rho_{B\,I\,O\,M\,A\,S\,S}\cdot Y_{i}}$$

where EC is expressed in kWh/kg_DW_, *C*_*p*_ is the specific heat of the aqueous algae suspensions (∼4.186 kJ/kg), *T*_0_ is the reference temperature (25°C), η*_*PUMP*_* is the overall efficiency of HPH pumping system (0.87) (Carullo et al., 2018), *ρ_*BIOMASS*_* is the density of microalgal suspensions (∼1000 kg/m^3^), 3600 is the conversion factor between kJ and kWh, and *Y*_*i*_ is the recovery yield (in kg/kg of DW microalgae) of the interest compounds (*i* = WSP, C-PC, CH) achieved upon the different extraction processes.

### Analytical Methods

#### Optical Microscopy and Scanning Electron Microscopy (SEM) Analysis

The morphological features and cellular details of untreated (control) and treated (PEF, HPH) algae cells were analyzed by using either optical or Scanning Electron Microscopy (SEM). In the first case, the microscopic images were acquired with an inverted optical microscope (Nikon Eclipse TE2000-S) at 20× magnification. For SEM analysis, pellets derived from the centrifugation of untreated and treated (PEF or HPH) algae suspensions were prepared as described by Carullo et al. (2018) and analyzed in a high-resolution ZEISS HD15 Scanning Electron Microscope (Zeiss, Oberkochen, Germany).

#### Proteins Analysis

The water-soluble proteins content of supernatants from untreated, PEF, and HPH treated samples was evaluated by using the method of Lowry et al. (1951), with some modifications as described elsewhere (Carullo et al., 2018). Specifically, the reactive system consisted in 0.5 mL of diluted (1/2, v/v in ultra-pure water) Folin-Ciocalteau reactive (Folin and Ciocalteau, 1927), to which 1 mL of fresh sample (supernatant), previously mixed with 5.0 mL of the reactive “C” [50 volumes of reactive “A” (2% Na_2_CO_3_ + 0.1 N NaOH) + 1 volume of reactive “B” (1/2 volume of 0.5% ⋅ CuSO_4_ 5H_2_O + 1/2 volume of 1% KNaC_4_H_4_O_6_ ⋅ 4H_2_O)] (Sigma Aldrich, Milan, Italy) were added. Absorbance was measured at 750 nm against a blank (5 mL reactive “C” + 1 mL deionized water + 0.5 mL Folin-Ciocalteau reactants), 35 min after the start of the chemical reaction, by using a V-650 Spectrophotometer (Jasco Inc., Easton, MD, United States). Bovine serum albumin (BSA) (A7030, Sigma Aldrich, Milan, Italy) was used as a protein standard. The protein yield (Y_WSP_) was expressed as:

(4) $$Y_{W\,S\,P}=\frac{C_{W\,S\,P,s\,u\,p}}{C_{W\,S\,P,b\,i\,o\,m\,a\,s\,s}}$$

where C_WSP__,sup_ is the protein content in the supernatant (% DW), and C_WSP,biomass_ is the total protein content on DW (% DW) achieved upon HPH treatment.

#### Carbohydrates Analysis

The total carbohydrates concentration of the supernatants was analyzed according to the phenol-sulfuric acid method previously described by DuBois et al. (1956). 0.2 mL of 5% (w/w) phenol and 1 mL of concentrated sulfuric acid (Sigma Aldrich, St. Louis, United States) were added to 0.2 mL of diluted supernatant (Dilution Factor = 5). Samples were then incubated at 35°C for 30 min before reading the absorbance at 490 nm against a blank of 0.2 mL 5% (w/w) phenol, 1 mL concentrated sulfuric acid, and 0.2 mL of deionized water. D-Glucose (G8270, Sigma-Aldrich, Milan, Italy) was used as a standard. The carbohydrate yield (Y_CH_) was expressed as:

(5) $$Y_{C\,H}=\frac{C_{C\,H,s\,u\,p}}{C_{C\,H,b\,i\,o\,m\,a\,s\,s}}$$

where C_CH,sup_ is the carbohydrates content in the supernatant (% DW) and C_CH,biomass_ is the total carbohydrates content on DW (% DW) achieved upon HPH treatment.

#### C-Phycocyanin (C-PC) and Purity Ratio

The quantification of C-PC content of the supernatants was performed according to the method of Bennett and Bogorad (1973), which is based on the measurements of absorbance (A) of the samples at two fixed wavelengths (λ_1_ = 615 nm, and λ_2_ = 652 nm). The C-phycocyanin concentration (C-PC), expressed as mg/g_DW_ of supernatant, was evaluated according to Eq. (6):

(6) $$C-P\,C=\frac{\left(A_{615\,n\,m}-0.474\cdot A_{652\,n\,m}\right)}{5.34\cdot C_{x}}$$

The C-PC yield (Y_*C–PC*_) was expressed as:

(7) $$Y_{C-P\,C}=\frac{C_{C-P\,C,s\,u\,p}}{C_{C-P\,C,b\,i\,o\,m\,a\,s\,s}}$$

where C_C–PC,sup_ is the C-PC content in the supernatant (% DW), and C_C–PC,biomass_ is the total C-PC content on DW (% DW) achieved upon HPH treatment.

The purity of C-PC extract was monitored spectrophotometrically and calculated by the Eq. (8) (Abelde et al., 1998; Martinez et al., 2017):

(8) $$E\,P=\frac{A_{615\,n\,m}}{A_{280\,n\,m}}$$

where EP is the protein extract purity, A_615 nm_ absorbance represents the maximum absorption of the C-phycocyanin peak, proportional to its concentration in the supernatant, and A_280 nm_ is the absorbance of the at 280 nm, indicating the total concentration of proteins in the supernatant.

### Statistical Analysis

All treatments and analyses were performed in triplicate and the results were reported as mean values ± standard deviations. The statistical analysis was performed with IBM SPSS Statistics 20.0 (SPSS Inc., Chicago, United States) software by means of One-way analysis of variance (ANOVA). Tukey’s test was executed at a fixed significance level (*p* ≤ 0.05), for the determination of any statistical difference among the untreated and processed samples.

## Results and Discussion

### Influence of PEF Treatment Intensity on the Recovery of Water-Soluble Intracellular Compounds

The biomass composition of *A. platensis* used in this study was quantified upon complete cell disruption by HPH treatment (*P* = 150 MPa; *n*_*P*_ = 3). Results revealed that the extractable content of WSP and CH from *A. platensis* cells was 68.5% DW and 15.8% DW, respectively, which are in line with the total content of proteins (55–70% DW) and carbohydrates (13–20% DW) typically reported for this cyanobacterium (Lupatini et al., 2016). At the same time, it was also found that the total content of C-PC was 5.7% DW, which is consistent with the content found in other works (Martinez et al., 2017; Jaeschke et al., 2019). The results of this study will be presented in terms of extraction yields expressed with respect to this biomass composition according to Eqs. (4–6).

**FIGURE 2:**
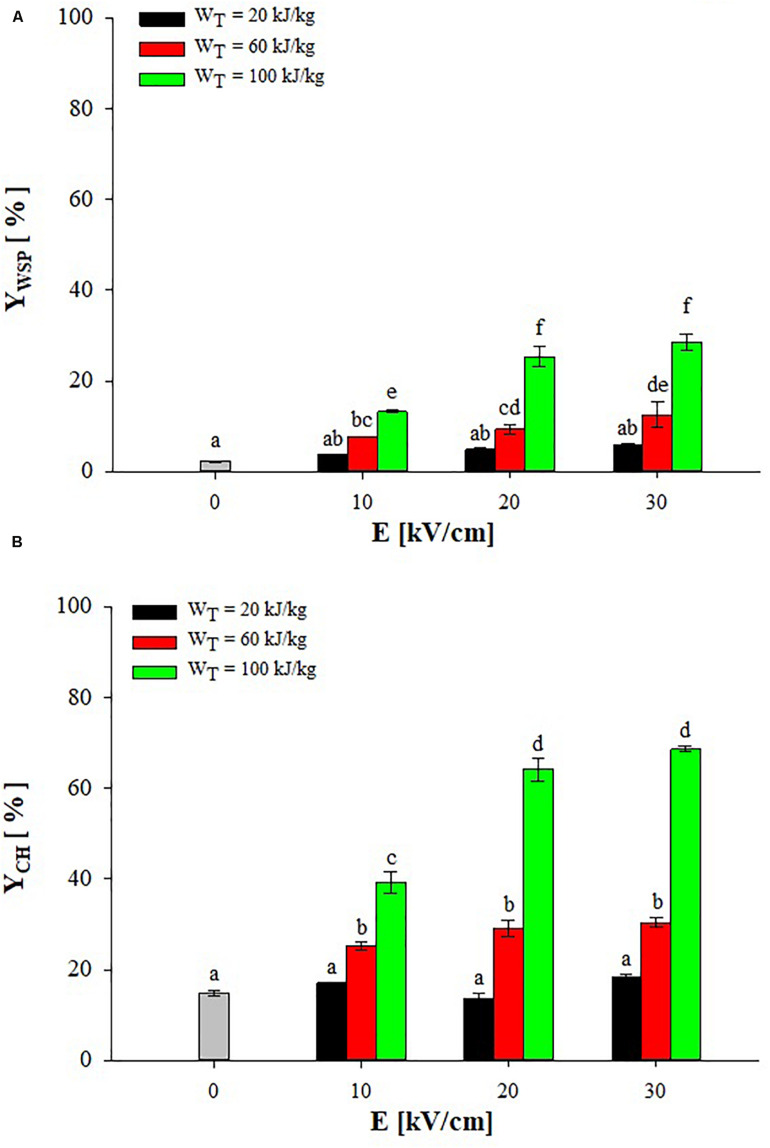
Extraction yield of water-soluble proteins **(A)** and carbohydrates **(B)** in the supernatant of untreated (0 kV/cm) and PEF (T_IN_ = 25°C) treated *A. platensis* suspension 3 h after treatment as a function of the field strength and for different energy input. The yields were calculated considering 100% extraction yield upon the HPH treatment (*P* = 150 MPa, *n*_*P*_ = 3). Different letters above the bars indicate significant differences among the mean values of the samples (*p* ≤ 0.05).

Figure 2 shows the extraction yields of water-soluble proteins (WSP) and carbohydrates (CH) detected in the supernatant of untreated and PEF-treated (monopolar pulses; T_IN_ = 25°C) *A. platensis* cell suspensions at different field strength (10–30 kV/cm) and energy input (20–100 kJ/kg_susp_), after 3 h of extraction in water.

Results show that a small leakage of water-soluble compounds from untreated algae cells occurred during the extraction step, leading to final yields of proteins and carbohydrates in the aqueous supernatant of 2.2 and 14.8%, respectively. This leakage of intracellular compounds may be ascribed to either a concentration gradient across the intact cell membranes or to spontaneous cell lysis (Carullo et al., 2018).

The exposure of *A. platensis* cell suspensions to an external electric field that caused the electroporation of cytoplasmatic membranes, thus facilitating the mass transfer of intracellular compounds toward the external medium, markedly enhanced the extraction efficiency up to 12.7-fold for WSP and 4.6-fold for CH, as compared with untreated samples. However, significant differences (*p* ≤ 0.05) between untreated and PEF-treated samples were detected only when energy input greater than 20 kJ/kg_susp_ were delivered to the algae suspension, independently of the field strength applied. This observation confirms the results obtained by other scientists on the electroporation of bacteria and microalgae (Garcia et al., 2007; Luengo et al., 2015; Martinez et al., 2017; Carullo et al., 2018; Jaeschke et al., 2019), indicating the key role played by the energy input, besides the electric field strength applied, in determining the degree of cell membrane permeabilization required to intensify the extractability of target intracellular compounds.

Moreover, it is worth noting that, among the PEF-treated samples, the applied field strength was likely high enough to induce the electroporation of the algae cells so that its effect appeared less important than that of the energy input within the investigated range, which is in agreement with previous findings (Pataro et al., 2017, 2019; ‘t Lam et al., 2017a; Carullo et al., 2018). In particular, significant differences (*p* ≤ 0.05) in the content of both intracellular compounds were detected when PEF treatments were carried out at different energy inputs, regardless of the field strength applied. As an example, when the energy input was increased from 60 to 100 kJ/kg_susp_ at 20 kV/cm, the content of proteins and carbohydrates in the supernatant increased by 2.7 and 2.2 times, respectively. In contrast, when PEF treatments were carried out at different field strengths, significant (*p* ≤ 0.05) increase in the content of both proteins and carbohydrates was detected only when the field strength was increased from 10 to 20 kV/cm and for a fixed energy input of 100 kJ/kg_susp_. At a fixed energy input of 60 kJ/kg_susp_, instead, a significant (*p* ≤ 0.05) increase was observed only for proteins when the field strength was increased from 10 to 30 kV/cm.

From the results of Figure 2, it can be concluded that a field strength of 20 kV/cm and an energy input of 100 kJ/kg_susp_ were sufficient to significantly intensify the extractability of proteins and carbohydrates from *A. platensis* cells, leading to extraction yields of 25.4 and 64.1% of biomass WSP and CH content, respectively.

The positive impact of PEF pre-treatment on the extraction of valuable intracellular compounds from *A. platensis* cell suspensions was also previously observed by other scientists (Aouir et al., 2015; Martinez et al., 2017; Jaeschke et al., 2019), even though, to date, only Jaeschke et al. (2019) focused on PEF-assisted extraction of total proteins, while no work was addressed to the extractability of carbohydrates from this cyanobacteria. However, these authors found a lower amount in WSP (4.84% DW biomass) as compared with that detected in the present work (17.4% DW biomass), despite the algae suspension was subjected to similar energy input (56–122 kJ/kg_susp_) but at higher field strength (40 kV/cm) than those used in our work. The differences in PEF equipment, experimental conditions, algae strain, and cultivation techniques could contribute to explain these different results.

Moreover, in agreement with previous research (Goettel et al., 2013; Postma et al., 2016; Pataro et al., 2017; ‘t Lam et al., 2017a; Carullo et al., 2018), results of the present work seem to highlight the capacity of PEF to efficiently release small components, such as CH and WSP of small molecular weight, while most proteins, which are likely larger and more bounded to intracellular structure, remained entrapped into cells after PEF treatment. This may be explained considering that, in comparison with HPH, PEF is a mild disruption technology able to simply permeabilize the cell membranes (Carullo et al., 2018), without affecting the rigid outer cell wall of most algae cells (‘t Lam et al., 2017b), thus limiting the mass transfer of some intracellular compounds.

In this line, differences in cell wall composition could in part also explain the higher extraction yields of protein (17.1% DW) achieved in this work after aqueous extraction of PEF-treated *A. platensis* cells (Figure 2), in comparison to those (1–13% DW) detected by other scientists for different microalgae species, such as *Chlorella vulgaris*, *Auxenochlorella protothecoides*, *Neochloris oleoabundans*, *C. reinhardtii* (Goettel et al., 2013; Postma et al., 2016; Pataro et al., 2017; ‘t Lam et al., 2017a,b; Carullo et al., 2018). It is known that *A. platensis* has a relatively fragile cell wall, composed mainly of murein (peptidoglycan) without any cellulose (Lu et al., 2006; Safi et al., 2014), while the other microalgae cited above have a more robust cell wall, mainly composed of cellulose and hemicelluloses, which would explain the lower protein extraction yield in these cases (Lu et al., 2006; Safi et al., 2015; Jaeschke et al., 2019).

According to the results shown so far, further investigations aimed at studying the influence of pulse polarity and mild heating on the extraction efficiency of valuable compounds from *A. platensis* cell suspensions were carried out with the PEF conditions set at 20 kV/cm and 100 kJ/kg_susp_.

### Effect of Pulse Polarity on the Recovery of Water-Soluble Compounds and Cell Morphology

**FIGURE 3:**
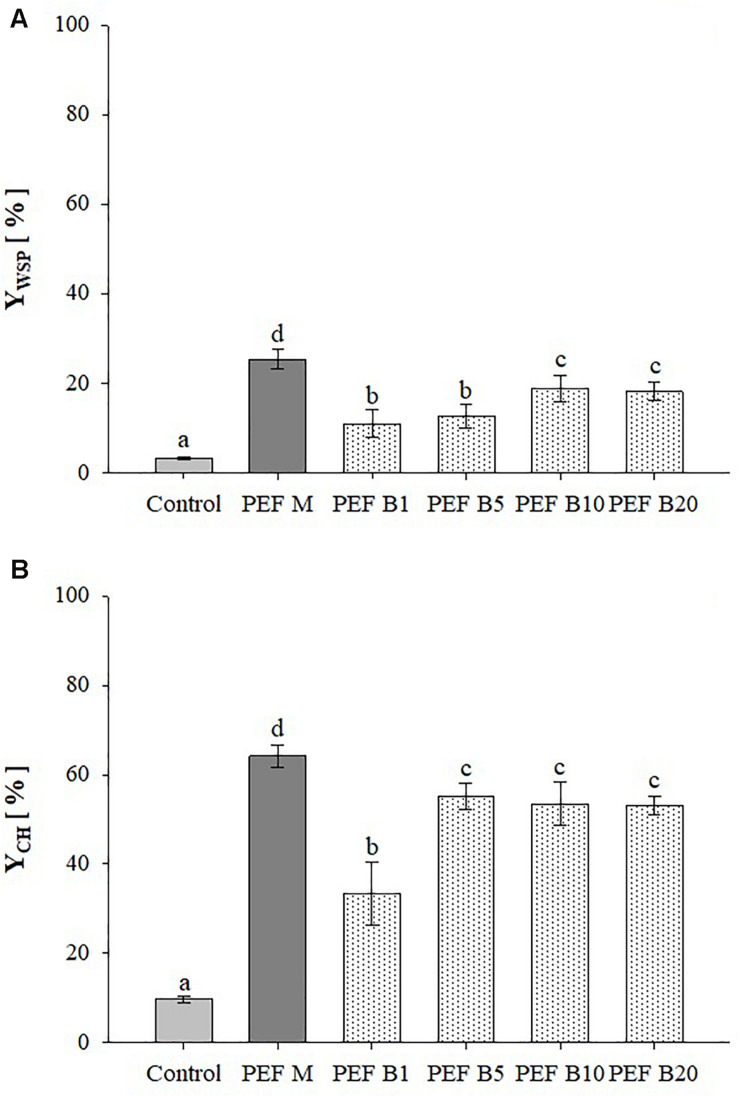
Extraction yield of water-soluble proteins **(A)** and carbohydrates **(B)** in the supernatant of untreated (Control) and PEF-treated (*E* = 20 kV/cm; *W*_T_ = 100 kJ/kg_susp_; T_IN_ = 25°C) *A. platensis* suspensions with either monopolar (M) or bipolar (B) pulses at variable delay time (1, 5, 10, and 20 μs). The yields were calculated considering 100% extraction yield upon the HPH treatment (*P* = 150 MPa, *n*_*P*_ = 3). Different letters above the bars indicate significant differences among the mean values (*p* ≤ 0.05).

Figure 3 shows the extraction yields of WSP and CH detected in aqueous extracts obtained from untreated and PEF treated *A. platensis* cell suspensions at constant treatment intensity (E = 20 kV/cm; W_T_ = 100 kJ/kg_susp_) and inlet temperature (T_IN_ = 25°C), using either monopolar pulses or bipolar pulses at variable delay times (1–20 μs). The results show that, regardless of the delay time, bipolar pulses significantly (*p* ≤ 0.05) increased the content of WSP and CH in the extracts, as compared with untreated samples. Interestingly, changing in pulse delaying times resulted in different extraction yields of the intracellular compounds of interest, showing the existence of a threshold delay time above which no additional positive effect was detected. In particular, significant differences (*p* ≤ 0.05) were observed only when the delay time was increased from 5 to 10 μs for proteins and from 1 to 5 μs for carbohydrates, which led to a maximum recovery yield of 18.8 and 55.2%, respectively.

To the best of our knowledge, the literature data lack sufficient information on the effect of bipolar pulses at different delay times on the extractability of valuable intracellular compounds from algae cells. However, when looking into microbial inactivation, Evrendilek and Zhang (2005) reported that changing pulse delaying times within the range 3–1430 μs resulted in different inactivation levels of *E. coli* O157:H7 inoculated in either apple juice or skim milk. In particular, the authors found that 20 μs was the most effective delay-time value within the investigated range, which appears somehow consistent with results achieved in the present study. The authors concluded that most probably, there was not enough time to charge capacitors of their pulse generator after each discharging when the duration of pulse delay time was really short (3–5 μs). On the other hand, as the delay time between pulses of opposite polarities was lengthened up to 1430 μs, the time interval between two pulses of opposite polarity was so long that adversely affected the efficacy of the treatment. This explanation could not justify our results due to the narrower range of delay time investigated and to the fact that the same voltage value was measured across the electrodes of the treatment chamber when bipolar pulses of different delaying times were delivered (data not shown).

As it is shown in Figure 3, the extracts obtained for the application of bipolar pulses showed significantly (*p* ≤ 0.05) lower yields of intracellular compounds of interest, as compared to the samples treated with monopolar pulses, being the extraction efficiency decreased by 1.4-fold for proteins and 1.2-fold for carbohydrates.

Only a few authors have investigated the comparative effect of mono ad bipolar pulses on the extent of cell membrane permeabilization of biological cells, but focusing only on microbial inactivation and obtaining controversial results. For example, Chang (1989) and Qin et al. (1994) found that bipolar pulses provided a more efficient inactivation of microorganisms in liquid foods, as compared to monopolar pulses. To explain these results the authors proposed that the alternating stress induced by bipolar pulses results in structural fatigue of the membrane, which thereby enhances its susceptibility to electrical breakdown. In contrast with these findings, Beveridge et al. (2002) observed that bipolar pulses did not provide superior inactivation levels of different bacterial cells compared with monopolar pulses. In another study, Evrendilek and Zhang (2005) found that there was no significant difference (*p* > 0.05) between mono and bipolar pulses on the inactivation of bacterial cells inoculated into apple juice, while bipolar pulses resulted significantly more efficient than their monopolar counterpart for the inactivation of the same bacteria in skim milk. In agreement with Qin et al. (1994), the authors explained this different behavior as due to the deposition of milk proteins on the electrode surface (fouling effect) when monopolar pulses were applied, which caused several problems such as distortion of the electric field within the treatment zone, thus lowering the PEF performance.

In the case of the results of Figure 3, the lower extractability of water-soluble molecules from *A. platensis* cells measured for bipolar pulses than for monopolar ones applied at the same intensity could be explained in term of the slightly lower efficacy in opening the pores at the cellular membrane level of the bipolar pulses. In particular, this explanation can be supported by the previously observed polarization behavior of the cell membranes when exposed to pulses of different polarity and delay time (Beveridge et al., 2002). In this regard, it can be hypothesized that when monopolar pulses of sufficient width and amplitude, such as those used in this work, are applied, a cumulative build-up of charges across the cell membrane occurs, and when the transmembrane potential threshold is exceeded, the membrane becomes electroporated, exhibiting increased permeability. On the other hand, when polarities switch in the bipolar pulses, an effect of residual polarization, due to the so-called cancelation or healing mechanism (Beveridge et al., 2002; Sweeney et al., 2016), may occur that would decrease the probability of membrane permeabilization. In such a case, it has been proposed that the polarization effect induced by the previous pulse would first have to be neutralized before reverse polarization of the membrane can occur when the second pulse of opposite polarity is applied. This effect could cause an incomplete charging of cell membranes, leading to the formation of a smaller number of pores or pores of smaller size in the cell membrane than those induced by monopolar pulses of the same intensity, lowering the extractability of intracellular compounds. However, such cancelation effect could be partially mitigated at the expense of either higher applied voltages or by increasing the time elapsing between two consecutive pulses of opposite polarity (Sweeney et al., 2016), as if they were delivered independently, which is consistent with results presented in Figure 3.

**FIGURE 4:**
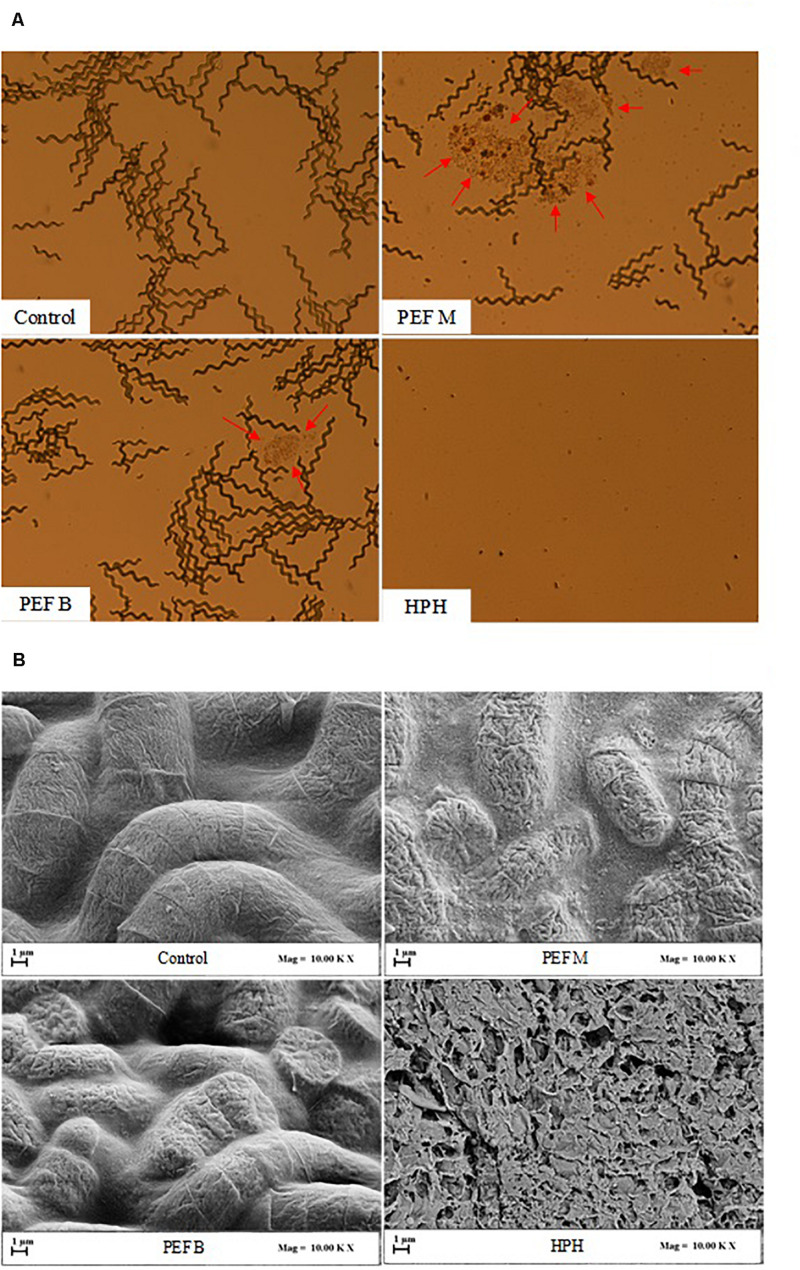
Optical microscopy (20× magnification) **(A)** and scanning electron microscopy (SEM) **(B)** of *A. platensis* cells, before (Control) and after PEF (*E* = 20 kV/cm; *W*_T_ = 100 kJ/kg_susp_; T_IN_ = 25°C) using either monopolar (PEF M) or bipolar (PEF B) pulses, and HPH (*P* = 150 MPa; *n*_*P*_ = 3) treatment. Red arrows indicate release of intracellular material.

The results of Figure 3 are also corroborated by the micrographs presented in Figure 4, which clearly show the different impact of HPH treatments and PEF treatments of different pulse polarity on the morphology of *A. platensis* cells. From the micrographs of Figure 4A it is possible to notice that, as expected, HPH caused the complete disruption of *A. platensis* cells and the formation of cell debris, which was consistent with the results of Figure 3. The application of a PEF treatment with monopolar pulses (PEF M) caused only the partial separation of trichomes that form the characteristic cylindrical filaments of *A. platensis*, and preserved the overall structure of the cells, avoiding the formation of cells debris. It is also worth noting that PEF M treatment induced the formation of colored spots surrounding the algae cells (red arrows), likely due to the leakage of intracellular matter into the extraction medium, which can be attributed to the formation of pores in the cell membranes of the algae cells. Similarly, Martinez et al. (2017) observed that, while a highly intensive cell disruption method, such as bead milling, caused the complete breakage of the *A. platensis* cells, PEF treatments carried out using monopolar pulses led only to a partial fragmentation of cells in trichomes, with no visible effects on the whole structure of the algae, but with a clear release of intracellular matter.

In contrast, the application of bipolar pulses (PEF B) evidenced a lower release of intracellular matters and a lower separation of cells in trichomes than monopolar pulses, thus revealing a lower capability of inducing electroporation phenomena, which is consistent with the results illustrated in Figure 3.

The results of Figure 4A are also supported by the corresponding SEM images shown in Figure 4B. The surface of untreated *A. platensis* cells appeared regular and smooth. Interestingly, the application of PEF with either monopolar (PEF M) or bipolar (PEF B) pulses led to an increase of surface roughness and the formation of cracks and depressions on the surface of the cells, more evident for the samples treated with monopolar pulses, which could be ascribed to the mentioned more intense electroporation phenomena and subsequent more abundant leakage of intracellular compounds. Similar findings were previously reported by Han et al. (2019), who observed an increase in surface roughness of *C. pyrenoidosa* cells after the electroporation effect induced upon the application of PEF treatment at 20 kV/cm. On the other hand, in agreement with the results of a previous research carried out on *C. vulgaris* microalgae (Carullo et al., 2018), the results of Figure 4B show that HPH treatment led to the formation of a large amount of cell debris, reflecting its capability of inducing full cell disruption (Figure 2).

### Effect of Combined PEF-Temperature Treatment on the Extractability of Water-Soluble Compounds

Processing temperature during PEF treatment, through the interaction with the electrical parameters, may influence the extent of membrane electroporation and the subsequent extraction of intracellular compounds from PEF-treated biomass. Therefore, the potential of PEF in a hurdle approach with mild heating was investigated to furtherly intensify the recovery yield of water-soluble compounds.

**FIGURE 5:**
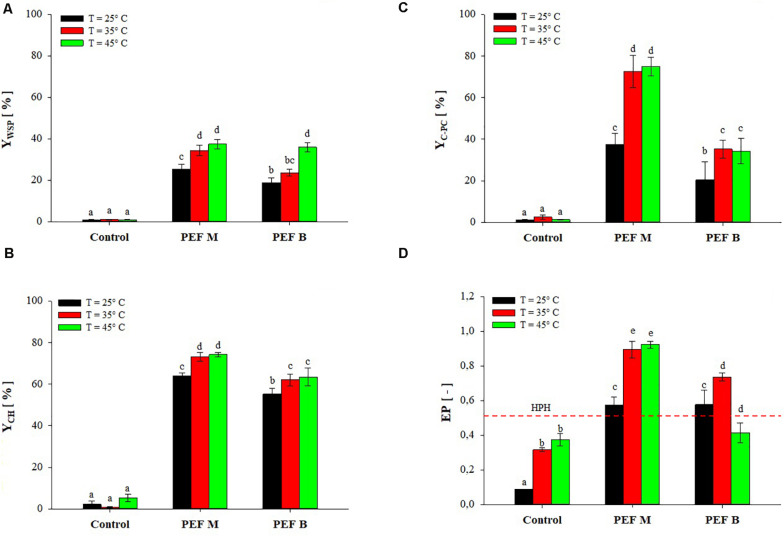
Extraction yield of water-soluble proteins **(A)**, carbohydrates **(B)**, and C-PC **(C)**, and protein purity **(D)** of extracts obtained from untreated (Control) and PEF-treated (*E* = 20 kV/cm; *W*_*T*_ = 100 kJ/kg_susp_) *A. platensis* suspensions with either monopolar (M) or bipolar (B) pulses (10 μs pulse delay time), as a function of the inlet processing temperature The yields were calculated considering 100% extraction yield upon the HPH treatment (*P* = 150 MPa, *n*_*P*_ = 3). Different letters above the bars indicate significant differences among the mean values (*p* ≤ 0.05).

Figure 5 shows the extraction yields of WSP, CH, and C-PC as well as the C-PC purity for untreated and PEF-treated (*E* = 20 kV/cm, *W*_*T*_ = 100 kJ/kg_susp_) *A. platensis* cell suspensions, using either monopolar and bipolar pulses, after mildly pre-heating the algae biomass at temperatures from 25 to 45°C.

The results show that only a small fraction of the total content of WSP (Y_WSP_ < 1.1%), CH (Y_CH_ < 5.2%), and C-PC (Y_C–PC_ < 2.5%) was released in the supernatant of the algae suspensions at a processing temperature between 25 and 45°C when no PEF treatment was applied. This suggests that no cell structural damage was induced by either pumping of *A. platensis* cell suspensions through the PEF plant or by mild heating. This is also corroborated by the findings of Jaeschke et al. (2019), who observed that pumping alone or thermal treatment at 42°C for 15 min of *A. platensis* cell suspensions were not sufficient to induce damages in the cell envelope and to extract appreciable amounts of WSP and C-PC, even after 6 h of incubation.

However, when PEF pre-treatments of different polarity were applied at processing temperature between 25 and 45°C, the amount of WSP (Figure 5A), CH (Figure 5B), and C-PC (Figure 5C) significantly (*p* ≤ 0.05) increased, as compared with untreated samples, in a manner dependent on pulse polarity, temperature and type of compounds to be extracted. In particular, the capability of PEF treatment at room temperature to induce cell membrane permeabilization, and the subsequent intensification of the extractability of WSP (25.4% for PEF M, 18.8% for PEF B) and CH (64.1% for PEF M, 55.2% for PEF B), has been also previously shown in Figures 2, 3. Coherently with these findings, the results of Figure 5C show that the application of PEF treatment at room temperature also caused a significant (*p* ≤ 0.05) improvement in the release of C-PC, as compared with the untreated samples, leading to an extraction yield of 37.4% for monopolar pulses and 20.4% for bipolar pulses.

However, when the processing temperature was raised to 35°C, a clear synergistic effect of the combined treatment was detected for monopolar pulses, leading to an increase in WSP, CH, and C-PC yields up to 34.4, 73.1, and 72.5%, respectively. At this processing temperature, a slight synergistic effect was also observed when bipolar pulses were applied which, however, still appeared less effective than monopolar pulses, leading to extraction yields of 23.7, 62.0, and 35.2% for WSP, CH, and C-PC, respectively.

Further increases of the processing temperature up to 45°C appeared to contribute to a significant (*p* ≤ 0.05) increase in the extraction yield, but only for WSP and when bipolar pulses were applied. It is worth noting that, in this latter case, the extraction yield of WSP increased up to 36.0%, a level comparable to that detected in samples treated with monopolar pulses (37.4%) at the same temperature.

The observed increase in the release of intracellular compounds with increasing of the inlet processing temperature up to 45°C during PEF treatment, was likely due to the interaction between field strength and temperature rather than further cellular damages induced by excessive heating of the samples. This is corroborated by the fact that the maximum sample temperature measured at the exit of the PEF chamber was about 51°C, which was measured under the most intensive treatment conditions (30 kV/cm, 100 kJ/kg) for an inlet temperature of 45°C. Additionally, the suspension was maintained at this temperature for only 2–3 s before being collected in plastic tubes and immediately placed in a water-ice bath.

The positive impact of the combined PEF-heat treatment on the extraction of valuable compounds from algae cell suspensions was previously observed by few other scientists, even though, to date, no previous works dealt with the use of bipolar pulses. For example, Postma et al. (2016) studied the effect of PEF treatment (17.1 kV/cm; 100 kJ/kg_susp_) combined with mild heating (25–65°C) on the extractability of valuable compounds from *C. vulgaris* cells suspensions. They observed only a slight positive interaction between PEF and temperature on the recovery of WSP when the temperature was increased in the range 25–45°C, leading to a maximum extraction yield of 4.4%. The analysis of the carbohydrates in the supernatant, instead, revealed the existence of a clear synergistic effect of the combined treatment, but only when the processing temperature was increased from 45 to 55°C, leading to an increase in carbohydrates yield from 25 to 39% of total biomass carbohydrate content. With regard to the extraction of pigments, Luengo et al. (2015) found that an increase of the temperature of PEF treatment (25 kV/cm, 150 μs) of *C. vulgaris* from 10 to 40°C increased the concentration of lutein in the extracts from 451 to 753 μg/g_DW_. Similarly, Martinez et al. (2017) found that the increment of the processing temperature from 10 to 40°C during PEF treatment (25 kV/cm, 150 μs) of *A. platensis* cells suspensions remarkably increased the extraction yield of C-phycocyanin up to a maximum value of 16.0% DW.

Interestingly, in all these works it was stated that mild heating of algae suspension contributes not only to enhance the diffusivity and solubility of intracellular compounds in water but also to make the lipid bilayer of the cell membrane more susceptible for breakdown under the PEF treatment (Timmermans et al., 2014; Luengo et al., 2015; Postma et al., 2016; Martinez et al., 2017). This would also explain the synergistic effect of the combined PEF-heat treatment on the extractability of water-soluble compounds observed in the present work.

However, although the hurdle approach revealed a positive interaction between electric field and moderate temperatures, the results of Figure 5 show that the maximum amount of the target compounds released after the combined treatment still remained lower than that achieved with the benchmark HPH process. This is somehow consistent with the findings previously reported by other scientists. For example, when comparing the extraction yield of water-soluble compounds achieved by the combined PEF-heat treatment of microalgae with those obtained after complete cell disruption by bead milling, Postma et al. (2016) estimated that about 10% of the total content of WSP was extracted from *C. vulgaris* cells, whereas Martinez et al. (2017) calculated a 70% of the total content of C-PC from *A. platensis* cells. This suggests that the combination of PEF with mild heating treatment is effective in the selective release of CH, C-PC, and WSP of small molecular weight compounds, while the extraction of larger compounds more bounded to intracellular structure, would require the application of more effective cell disruption techniques than PEF, such as bead milling or HPH (Postma et al., 2016; Martinez et al., 2017; Pataro et al., 2017; Carullo et al., 2018). In this latter case, however, higher recovery yields would likely be achieved at expenses of lower purity of the extracts. This is clearly shown by the results presented in Figure 5D, which highlights that, regardless of the processing temperature and pulse polarity, the purity of the C-PC extract from *A. platensis* cells suspensions upon the application of PEF pre-treatment was always higher (0.71–0.89) than that estimated for the extracts obtained after treating the cells with HPH (0.53), which is consistent with the finding of Martinez et al. (2017). Interestingly, since a C-PC purity of 0.7 is required for food-grade products (Vernès et al., 2015), the C-PC extract obtained after PEF treatment could potentially be used for food application without the need for further refining stages (Rito-Palomares et al., 2001).

### Energy Efficiency Analysis

After demonstrating that PEF treatment (20 kV/cm, 100 kJ/kg_susp_), especially when using monopolar pulses combined with moderate heating (35°C), is effective for promoting the selective extraction of water-soluble compounds from *A. platensis* cells suspensions, in the last step of this work we also evaluated its feasibility in terms of energy consumption.

Results of Table 1 highlight the comparison among the different cell disruption techniques investigated (PEF M and PEF B at processing temperature of 25 or 35°C, mild heating at 35°C, combined PEF (mono or bipolar)-heating (35°C), and HPH) in terms of the energy consumed to extract 1 kg of the target water-soluble compounds (WSP, CH, C-PC) from *A. platensis* cells. Overall, the estimated energy consumptions show that PEF is more efficient than HPH in a manner dependent on the type of compounds to be extracted and processing conditions applied. In particular, PEF M applied at room temperature enabled the recovery of either CH and C-PC at comparable yields with HPH, but with higher purity and significantly lower energy consumption, with the perspective of facilitating purification operations in downstream processes. In the case of WSP, instead, PEF M at room temperature showed only a slightly higher energy efficiency than HPH, likely due to the limited capacity of the simply electroporated cells to release a significant amount of large molecular-weight proteins in comparison with the cells completely disintegrated by HPH treatment. As expected, the lower extraction yields of WSP and C-PC achieved upon the application of PEF B at room temperature than PEF M, required higher energy consumption for the recovery of these water-soluble compounds. In comparison with HPH, PEF B was more energetically efficient only in the case of CH recovery.

**TABLE 1:** Specific energy consumptions (EC, in kWh/kg_DW_) of the different cell disruption techniques for the recovery of a unit mass of target compounds (WSP, water-soluble proteins; C-PC, C-phycocyanin; CHO, carbohydrates) from *A. platensis* microalgae suspensions.

**Disruption method**	**Specific energy consumption (kWh/kg_DW_)**		
	**WSP**	**C-PC**	**CH**
PEF M (T_IN_ = 25°C)	8.1	65.7	13.7
PEF B (T_IN_ = 25°C)	10.8	120.1	15.9
Mild heating (T_IN_ = 35°C)	79.6	401.3	443.9
Mild heating (T_IN_ = 35°C) + PEF M	8.4	47.9	17.1
Mild heating (T_IN_ = 35°C) + PEF B	12.1	100.1	20.1
HPH	10.5	110.5	53.7

*PEF M and PEF B indicate the PEF treatment (20 kV/cm, 100 kJ/kg) with mono and bipolar pulses (delay time = 10 μs), respectively. HPH treatment conditions: P = 150 MPa; n_*P*_ = 3.*

The mild heating of the algae suspension at 35°C without any PEF treatment scarcely affected the release of the target water-soluble compounds, requiring the highest expenditures of energy. Interestingly, the combined PEF M-temperature treatment required comparable energy consumptions per unit mass of WSP and CH to those required by PEF M at room temperature, while showing a significantly lower energy consumption for the recovery of C-PC. These results can be explained by the synergistic effect of the combined treatment on the extractability of intracellular compounds. A similar trend was observed when PEF B was combined with moderate heating, which appeared more energetically efficient than HPH for the recovery of only CH and C-PC.

These results are consistent with findings previously reported by other scientists when comparing PEF and HPH in terms of energy consumed to extracts water-soluble compounds, even though from different algae cells and focusing only on monopolar pulses during treatment at room temperature. For example, when Grimi et al. (2014) and Carullo et al. (2018) compared PEF and HPH in terms of the energy consumed to extract 1 kg of carbohydrates or proteins from *Nannochloropsis* spp. and *C. vulgaris* microalgae, they found that PEF was slightly less energetically efficient than HPH for the recovery of WSP, while showed lower energy consumption than HPH for the recovery of CH.

Further studies are required to comparatively investigate the effect of PEF and HPH on microalgae strain characterized by different cell envelops, as well as processing biomass with higher solid concentrations than the diluted suspension used in this work, since those parameters could significantly affect the energy efficiency of both PEF and HPH treatment (Goettel et al., 2013; Yap et al., 2015).

## Conclusion

Results obtained in this study have demonstrated the potential of PEF technology to intensify the selective release of water-soluble compounds (WSP, CH, C-PC) from *A. platensis* cell suspensions.

The extraction efficiency of intracellular compounds by PEF treatment depended on electrical parameters such as electric field strength, energy input, pulse polarity, and delay time between pulses of opposite polarity. The application of bipolar pulses resulted to be less effective in the permeabilization of the membranes of algae cells and the subsequent recovery of the target intracellular compounds than the usage of monopolar pulses, at least in the range of operative conditions investigated in this work. However, the optimization of the time delay between two consecutive pulses of opposite polarity appeared crucial to increase the probability of membrane permeabilization and the extraction efficiency of bipolar pulses. Therefore, further studies are needed in order to better elucidate the effect of bipolar pulses and the role played by the delay time and pulse width on the algae cell permeabilization. Improving the efficacy of bipolar pulses in the electroporation process could be very interesting, since its use will enable to drastically limit the occurrence of undesired electrochemical reactions (e.g., corrosion, electrolysis) at the electrode interface when monopolar pulses are applied (Pataro and Ferrari, 2020).

Interestingly, the use of PEF in a hurdle approach with mild heating of the biomass at 35°C remarkably enhanced the extractability of intracellular compounds showing a clear synergistic effect, regardless of the pulse polarity. In comparisons with the highly disruptive effect of HPH treatment, PEF enabled the selective release of low molecular weight WSP, CH and especially the recovery of high purity (>0.7) C-PC extract, lowering the energy consumption and without the formation of cell debris, which might facilitate the subsequent downstream purification operations.

## Data Availability Statement

All datasets generated for this study are included in the article/supplementary material.

## Author Contributions

GP and GF contributed to the conception and design of the study. DC was in charge of performing the chemical and statistical analysis and wrote the first draft of the manuscript. GP, FD, and DC performed the experiments. GF supervised the study. All authors contributed to manuscript revision, read and approved the submitted version.

## Conflict of Interest

The authors declare that the research was conducted in the absence of any commercial or financial relationships that could be construed as a potential conflict of interest.
